# Functional compartmentalization of Rad9 and Hus1 reveals diverse assembly of the 9‐1‐1 complex components during the DNA damage response in *Leishmania*

**DOI:** 10.1111/mmi.13441

**Published:** 2016-07-19

**Authors:** Jeziel D. Damasceno, Ricardo Obonaga, Elaine V. Santos, Alan Scott, Richard McCulloch, Luiz R. O. Tosi

**Affiliations:** ^1^ Department of Cell and Molecular BiologyRibeirão Preto Medical School, University of São PauloRibeirão Preto, SP14049‐900Brazil; ^2^ The Wellcome Trust Centre for Molecular Parasitology, Institute of Infection, Immunity and Inflammation, University of Glasgow; 120 University PlaceGlasgowG128TAUK

## Abstract

The Rad9‐Rad1‐Hus1 (9‐1‐1) complex is a key component in the coordination of DNA damage sensing, cell cycle progression and DNA repair pathways in eukaryotic cells. This PCNA‐related trimer is loaded onto RPA‐coated single stranded DNA and interacts with ATR kinase to mediate effective checkpoint signaling to halt the cell cycle and to promote DNA repair. Beyond these core activities, mounting evidence suggests that a broader range of functions can be provided by 9‐1‐1 structural diversification. The protozoan parasite *Leishmania* is an early‐branching eukaryote with a remarkably plastic genome, which hints at peculiar genome maintenance mechanisms. Here, we investigated the existence of homologs of the 9‐1‐1 complex subunits in *L. major* and found that LmRad9 and LmRad1 associate with chromatin in response to replication stress and form a complex *in vivo* with LmHus1. Similar to LmHus1, LmRad9 participates in telomere homeostasis and in the response to both replication stress and double strand breaks. However, LmRad9 and LmHus1‐deficient cells present markedly opposite phenotypes, which suggest their functional compartmentalization. We show that some of the cellular pool of LmRad9 forms an alternative complex and that some of LmHus1 exists as a monomer. We propose that the diverse assembly of the *Leishmania* 9‐1‐1 subunits mediates functional compartmentalization, which has a direct impact on the response to genotoxic stress.

## Introduction

Preservation and transmission of the eukaryotic genome rely on the cell's ability to detect and repair DNA damage. Thus, an extensive network of pathways coordinates DNA damage sensing, cell cycle progression and DNA repair processes. The Rad9‐Rad1‐Hus1 (9‐1‐1) heterotrimeric complex is a central player in the DNA Damage Response (DDR) of eukaryotic cells. The ring‐shaped 9‐1‐1 complex is structurally related to the PCNA clamp that acts in DNA replication and is loaded onto DNA during the early steps of the DDR (Bermudez *et al*., 2003). Initial characterization of the 9‐1‐1 complex was focused on its involvement in the cellular response to replication stress. Upon disruption of the replication reaction, integrity of the DNA molecule is endangered by the accumulation of single stranded DNA (ssDNA) stretches, and the response to these structures involves their recognition and binding by the Replication Protein A complex (RPA). RPA‐coated ssDNA facilitates the independent recruitment of both the 9‐1‐1 clamp and the ATR‐ATRIP kinase complex, which signals cell cycle arrest through the activation of the checkpoint kinase 1 (Zou and Elledge, 2003; Zou *et al*., 2003; Xu *et al*., 2008). In this context, 9‐1‐1 stabilizes the association of ATR‐ATRIP with the damaged DNA site and reinforces the checkpoint signal (Medhurst *et al*., 2008).

It is notable that the 9‐1‐1 structure provides three specific binding surfaces, suggesting functional diversification of the complex relative to the homotrimeric PCNA clamp. In fact, expanding evidence indicates a broad range of functions for the 9‐1‐1 complex besides its involvement in the replication stress response. For instance, yeast 9‐1‐1 is required for proper processing of DNA double strand break (DSB) ends (Ngo and Lydall, 2015), and human 9‐1‐1 has a role in DNA repair events as a ligand and modulator of enzymes from the Base Excision Repair pathway (Hwang *et al*., 2015). The clamp subunits have also been implicated in telomere homeostasis in yeast (Nakamura *et al*., 2002), nematodes (Ahmed and Hodgkin, 2000; Hofmann *et al*., 2002), and mammals (Francia *et al*., 2006). It is also becoming clear that functional diversification of 9‐1‐1 has been further intensified during evolution by the appearance of variants of Rad9 and Hus1. In mammals, the Rad9B paralog seems to be involved in cell cycle progression in the G1/S transition and also in the regulation of meiosis (Perez‐Castro and Freire, 2012, Lyndaker *et al*., 2013a, 2013b). In yeast, a Rad9 isoform participates in the response to heat shock stress in a 9‐1‐1 independent manner (Janes *et al*., 2012).

Considering the mounting evidence for functional diversification of the 9‐1‐1 complex, studying its structure and function in early‐branching eukaryotes provides an opportunity to understand the evolution of the DDR in the eukaryotic lineage and the breadth of DNA metabolic processes that 9‐1‐1 can act in. To date, no extended functional characterization of a putative 9‐1‐1 complex has been performed in protozoa. We have previously reported the identification of Rad9 and Hus1 homologs in *Leishmania*, a single‐celled kinetoplastid parasite, and presented evidence that LmHus1 plays a role in the replication stress response and cell cycle progression in *Leishmania major* (Nunes *et al*., 2011; Damasceno *et al*., 2013). Although putative homologs of Rad1 in kinetoplastids have been identified (MacNeill, 2014), no functional analysis has been reported. Considering the variety of roles for 9‐1‐1 in eukaryotic genome maintenance, we set out to investigate the formation and functioning of a 9‐1‐1 homolog in *Leishmania*, where surprising tolerance of genome variation has been reported, potentially indicating that genome maintenance mechanisms are peculiar. For instance, hallmarks of an unstable genome, such as gene amplification and chromosome copy number variation, are observed with considerable frequency in *Leishmania*, both in the lab and in wild isolates of the parasite. How the remarkable genome plasticity of this protozoan is generated and tolerated is poorly understood, though genome‐wide amplification events driven by repeated sequences and recombination have been documented (Beverley, 1991; Rogers *et al*., 2011; Ubeda *et al*., 2014). Thus, dissecting the structure and function of the *Leishmania* 9‐1‐1 complex may contribute to not only a better understanding of eukaryotic genome maintenance mechanisms, but also the strategies used by this parasite to overcome DNA injuries and to adapt to its environment.

In this report we demonstrate that the *L. major* 9‐1‐1 subunits LmRad9, LmRad1 and LmHus1 form a complex within the cell and associate with chromatin in response to replication stress. We also detail that LmRad9 participates in telomere homeostasis and that LmRad9 and LmHus1 are required for an effective response to both replication stress and DSBs. Despite these overlapping activities, we also demonstrate that LmRad9 and LmHus1 can be found outside the 9‐1‐1 complex and, consistent with this, deficiency in the genes leads to differing repair phenotypes. We take these findings as evidence that at least two of the *Leishmania* 9‐1‐1 subunits have evolved to perform compartmentalized genome maintenance functions.

## Results

### *L. major* expresses a 9‐1‐1‐homolog complex

**Figure 1:**
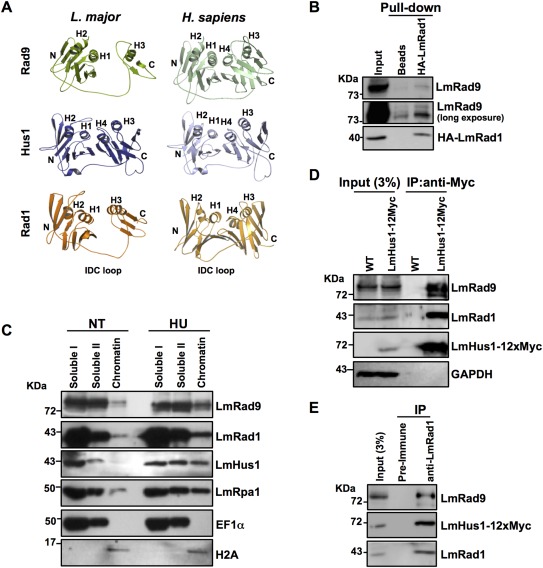
LmRad9, LmRad1, and LmHus1 form a complex *in vivo*. A. Ribbon diagram of the predicted model for Rad9, Rad1 and Hus1 homologs from *L. major* (left panels) as compared with the structure of 9‐1‐1 subunits from *H. sapiens* (right panels); α‐helices are indicated as H1 to H4; C and N indicate the globular domains containing the carboxyl‐ and amino‐terminus, respectively; structural prediction of LmRad9, LmRad1, and LmHus1 was performed with Phyre2 (http://www.sbg.bio.ic.ac.uk/phyre2/); images for each molecular model were prepared using PyMol (http://www.pymol.org/); images of human 9‐1‐1 was generated with the PDB file 3GGR. B. *In vitro* translated HA‐LmRad1 was used as bait in a pull‐down assay; total protein extract from *L. major* was incubated with beads only (lane indicated as beads) or with HA‐LmRad1 coupled to beads attached to anti‐HA antibody (lane indicated as HA‐LmRad1); the pulled down material was analyzed by western blot using anti‐HA and anti‐LmRad9 antibodies. C. LmRad1 overexpressor cells were left untreated (NT) or treated with 5 mM HU for ∼10 h and then subjected to fractionation; fractions corresponding to first and second round of extraction with Extraction Buffer (see methods for details) are indicated as Soluble I and Soluble II, respectively; fractions corresponding to the material released by DNAseI treatment are indicated as chromatin; fractions were analyzed by western blot with anti‐LmRad9, anti‐LmRad1 and anti‐LmHus1 antibodies; LmRpa1 was used as a positive control for chromatin binding upon HU treatment; EF1a was used as a marker for soluble proteins‐containing fraction; H2A was used as a marker for chromatin‐containing fractions. D. Extract from WT and LmHus1‐12xMyc‐expressing cells was subjected to immunoprecipitation (IP) with anti‐Myc antibody; IP products were analyzed by western blot with anti‐LmRad9, anti‐LmRad1 and anti‐Myc antibodies; the membrane was also probed with anti‐GAPDH antibody as a loading control. E. Extract from LmHus1‐12xMyc‐expressing cells was subjected to immunoprecipitation (IP) with pre‐immune or anti‐LmRad1 serum; IP products were analyzed by western blot with anti‐LmRad9, anti‐Myc and anti‐LmRad1antibodies.

We have previously reported that *L. major* homologs of Hus1 and Rad9 are expressed and form a complex *in vivo* (Damasceno *et al*., 2013). Our attempts to identify a Rad1 homolog using primary sequence homology searches were unsuccessful, suggesting that a possible Rad1 homolog in this parasite is highly diverged from its mammal or yeast counterparts, as revealed by MacNeill (2014). To circumvent this divergence, we used the BackPhyre approach (Kelley *et al*., 2015), in which the protein tertiary structure is used in the search for homologs. Our survey, which was conducted independently of the study by MacNeill (2014), also returned the *L. major* ORF LmjF.20.0390, which encodes a putative 362‐amino acid protein (hereafter referred as LmRad1) that presents ∼21% identity with the human Rad1 at the primary sequence level, and is phylogenetically related to Rad1 homologs from other eukaryotes (Supporting Information Figure S1). As presented in Fig. 1A, structure predictions of LmRad1 rendered a model with ∼99.5% confidence that reveals overall conservation of Rad1 structural characteristics, such as the globular amino and carboxyl domains connected by the Inter Domain Connecting (IDC)‐loop. Similar to what we found for LmRad9, but different from LmHus1, most of the conservation in LmRad1 was confined to the amino‐terminal region, whereas the carboxy‐terminus presented a considerably more diverged structure.

To investigate if LmRad1 could be part of a *L. major* 9‐1‐1 complex, we initially performed a pull‐down assay, using an *in vitro* translated hemagglutinin‐tagged version of LmRad1 (HA‐LmRad1) as bait. The western blot analysis presented in Fig. 1B demonstrates that LmRad9 was enriched in the samples that were pulled‐down with HA‐LmRad1. This result suggests that LmRad1 and LmRad9 can interact with each other. To investigate the involvement of LmRad1 and LmRad9 in DNA metabolism we analyzed their association with chromatin in response to genotoxic stress. We performed cell fractionation using cells exposed to the replication stalling agent hydroxyurea (HU). Western blot analysis (Fig. 1C) showed that, similar to LmHus1, LmRad9 and LmRad1 are significantly enriched in the chromatin fraction following exposure to HU. This result indicates that the 9‐1‐1 homolog subunits associate with chromatin in response to genotoxic stress.

To further test the association of LmRad1 with 9‐1‐1 subunits *in vivo* we performed co‐immunoprecipitation assays (coIP) using a cell line expressing a C‐terminally 12xMyc tagged version of LmHus1 (LmHus1‐12xMyc) (Supporting Information Figure S2). We used anti‐Myc antibody to immunoprecipitate LmHus1‐12xMyc and, as presented in Fig. 1D, western blot analysis revealed that both LmRad9 and LmRad1 were co‐precipitated with LmHus1‐12xMyc. We also used the same cell line in a coIP experiment using anti‐LmRad1 serum. As shown in Fig. 1E, western blot analysis revealed that both LmRad9 and LmHus1‐12xMyc were co‐precipitated with LmRad1. Taken together, these data indicate that LmRad9, LmRad1, and LmHus1 form a complex *in vivo*.

### LmRad9 is an essential gene in *L. major*

**Figure 2:**
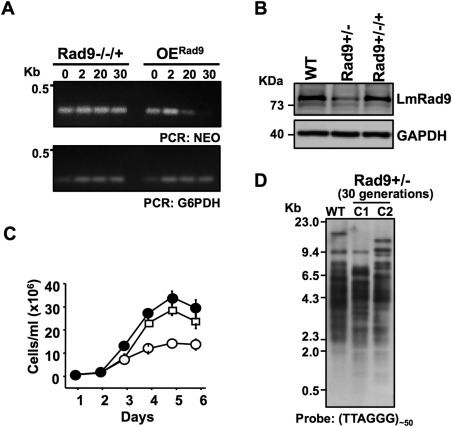
LmRad9 is an essential gene and its deficiency impairs proper cell proliferation and telomere maintenance. A. Detection of NEO by semi‐quantitative PCR using genomic DNA from LmRad9−/−/+ and OE^Rad9^ cell lines cultivated in the absence of G418 for the numbers of passages indicated at the top of each lane; PCR amplification of G6PDH was used as control. B. Western blot analysis of total cell extracts from WT, LmRad9+/− and LmRad9+/−/+ cell lines using anti‐LmRad9 antibodies; the same membrane was also probed with anti‐GAPDH as a loading control. C. Comparison of the growth pattern of WT (closed circles), LmRad9+/− (open circles) and LmRad9+/−/+ (open squares) cells. D. Southern blot analysis of telomeric sequence‐containing fragments generated by digestion of genomic DNA with the restriction enzymes *Cvi*QI, *Hpa*II, *Alu*I, and *Hha*I; genomic DNA from WT and two different clones (C1 and C2) of LmRad9+/− cells (after 30 generations) were analyzed.

We have previously reported that LmHus1 deficiency interferes with the maintenance of telomeres and impairs a proper response to genotoxic stress (Damasceno *et al*., 2013). Considering that LmHus1 and LmRad9 are two parts of a complex *in vivo* (Fig. 1D and E), we sought to compare the effects of LmRad9 and LmHus1 deficiencies. Our efforts to generate an LmRad9‐null cell line were unproductive: in common with what we previously observed for LmHus1, attempts to replace both LmRad9 alleles by sequential introduction of different drug resistance markers were unsuccessful and only selected heterozygous cell lines in which one allele was replaced. These findings indicate that both LmRad9 and LmHus1 are essential genes for *L. major* survival. In support of this, replacement of both LmRad9 alleles was possible after transfection of an episomal vector carrying a copy of the gene. A fluxogram illustrating the protocol for the generation of the different LmRad9 mutant cell lines is presented in Supporting Information Figure S3. We have tested LmRad9 essentiality using a typical approach to investigate the segregational loss of a complementing episome (Murta *et al*., 2009). Thus, cells in which both LmRad9 alleles had been disrupted while bearing an episomal copy of LmRad9 (LmRad9−/−/+) were cultivated in the absence of G418 to determine the stability of the episomal LmRad9‐expressing construct (pXG1‐LmRad9). As a control for the dynamics of episome loss we used an over‐expressor cell line (OE^Rad9^; Supporting Information Figure S3), which also carries pXG1‐LmRad9, but in which both genomic alleles of LmRad9 are intact. As presented in Fig. 2A, OE^Rad9^ cells had no detectable pXG1‐LmRad9 molecules after 30 passages (∼200 cell divisions) as monitored by semi‐quantitative PCR analysis. In contrast, the LmRad9−/−/+ cells did not show any significant loss of the pXG1‐LmRad9 signal over the same number of passages, which suggests that LmRad9 is essential for survival.

### LmRad9 deficiency impairs cell proliferation and telomere maintenance

Since we were not able to obtain LmRad9 null cells, we used the LmRad9+/− cell line, in which one allele was deleted, to investigate the function of this protein. As presented in Fig. 2B, LmRad9 levels were reduced to ∼50% in the LmRad9+/− cell line when compared with WT cells, demonstrating that we successfully generated a LmRad9‐deficient cell line (and consistent with the detectable loss of one allele; Supporting Information Figure S3). Growth profile analysis of this cell line revealed a significant defect when compared with WT cells (Fig. 2C), indicating that LmRad9 deficiency impairs cell proliferation. Moreover, addition of the episome expressing LmRad9 to these cells (LmRad9+/−/+; Fig. 2B and Supporting Information Figure S3) restored protein levels to that seen in WT (Fig. 2B) and led to a significant reversion of the growth defect (Fig. 2C). These findings indicate that the defective cell proliferation phenotype was specifically caused by LmRad9 deficiency.

To further investigate the role of LmRad9 in genome stability in *L. major* we compared the telomere length profile of the LmRad9+/− and WT cells. The 9‐1‐1 complex is required for telomere homeostasis in other eukaryotes (Hofmann *et al*., 2002; Nakamura *et al*., 2002; Francia *et al*., 2006) and we have previously reported that LmHus1 is required for telomere maintenance in *L. major* (Damasceno *et al*., 2013). The Southern blot analysis shown in Fig. 2D revealed a detectable increase in the abundance of fragments smaller than 2 kb in the two clones of LmRad9+/− tested. Also, the larger telomere‐containing fragment seemed to be either lost (C1) or shortened (C2). We did not observe further reduction on the size of telomere‐containing fragments up to 150 generations (Supporting Information Figure S4), indicating that the minimal telomere length was reached before 60 generations. These data demonstrate that telomeres shorten in LmRad9+/− cells, suggesting that LmRad9 is required for effective telomere maintenance in *L. major*.

### LmRad9 deficiency impairs cell cycle progression

**Figure 3:**
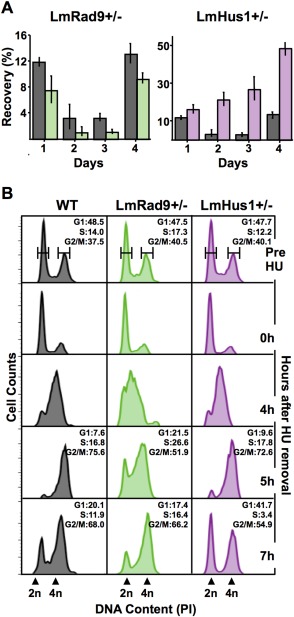
LmRad9 and LmHus1 have distinct roles in the response to replication stress. A. WT (gray), LmRad9+/− (green) and LmHus1+/− (purple) cells were treated with 10 mM HU for ∼15 h and seeded in drug‐free media at 10^5^ cells/ml; cell densities were assessed daily and recovery was calculated as a percentage of proliferation as compared with the nontreated cells; vertical lines on top of each bar indicate standard deviation. B. Cell cycle progression analysis of WT, LmRad9+/− and LmHus1+/− cell lines; cell cycle were blocked with 5 mM HU for 8 h, seeded in HU‐free medium and collected at the indicated time points; DNA content was examined by flow cytometry; each histogram represent data from 10,000 events; 2n and 4n indicate nonreplicated and replicated DNA, respectively; percentage of cells in gated G1, S, and G2/M phases is indicated for cells before HU treatment (Pre HU) and for cells at 5 and 7 h after HU removal.

To further understand the roles of LmRad9 in genome maintenance, we next investigated the effect of LmRad9 deficiency in the response to genotoxic stress. For this, we compared the growth recovery of LmRad9+/− and WT cells after being exposed to HU. As shown in Fig. 3A, the recovery of LmRad9+/− cells was significantly reduced when compared with WT cells. Similar results were observed when cells were exposed to other replication stress agents, such as the topoisomerase I inhibitor camptothecin (CPT) and the DNA methylating agent methyl methanesulfonate (MMS) (Supporting Information Figure S5). In contrast, the response of LmHus1+/− cells to each form of genotoxic stress was the opposite of the LmRad9‐deficient cells, with a considerably improved recovery when compared with WT cells (Fig. 3A and Supporting Information Figure S5). These findings are consistent with LmHus1+/− cells having an impaired ability to arrest cell proliferation in response to HU, CPT or MMS (Damasceno *et al*., 2013). Taken together, the growth analysis suggests that both LmRad9 and LmHus1 play a pivotal role in the control of cell proliferation upon replication stress, though the observed phenotypes indicate that the two factors can act through distinct mechanisms.

To further explore the differential modes of action for LmRad9 and LmHus1, we analyzed the cell cycle progression of LmRad9+/− and LmHus1+/− cells after release from HU treatment, which arrests *Leishmania* at the G1/S transition. DNA content was determined by flow cytometry analysis of cells collected at different time points after HU removal (Fig. 3B), revealing that LmRad9+/− cells were slower to progress through S phase than WT, since the proportion of cells in G1 and S phases was notably high at 5 h after HU removal, when most WT cells were in G2/M. Moreover, LmRad9 deficient cells presented a detectable reduction (relative to WT) in the proportion of the population that had returned to G1 ∼7 h after HU removal. These data are consistent with the delayed growth recovery of LmRad9+/− cells, and indicate that LmRad9 is required to promote cell cycle progression following replication stress, possibly acting in S phase. As previously reported, LmHus1 deficiency is linked to a deregulated progression through the cell cycle upon replication stress (Damasceno *et al*., 2013). In Fig. 3B, we confirm these findings, since the LmHus1+/− cells progressed more quickly through S and G2/M phases than WT cells, especially ∼7h after release from HU. Indeed, data presented in Fig. 3 reinforces the pronounced difference in response to HU of LmRad9+/− and LmHus1+/− cells, supporting the proposal that LmRad9 and LmHus1 can act through distinct mechanisms following the activation of the replication stress response in *Leishmania*.

**Figure 4:**
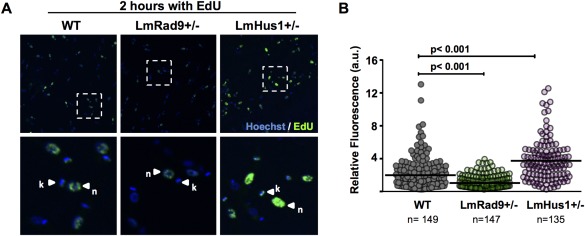
LmRad9 and LmHus1 deficiencies affect DNA synthesis. A. WT, LmRad9+/− and LmHus1+/− cells were incubated under the same conditions with 10 μM EdU for 2 h; the DNA was stained with Hoechst; bottom panels show enlarged images from the delimited regions in the upper panels; nuclear (n) and kinetoplastid (k) DNA are indicated by arrowheads. B. Graphical representation of EdU incorporation data as measured in arbitrary units (a.u.); each dot represent fluorescence intensity of an individual EdU‐positive cell; horizontal bars indicate the average fluorescence intensity; n indicates the number of EdU‐positive cells analyzed for each cell line; *p* value as determined by Kruskal–Wallis test is indicated.

The flow cytometry analysis above revealed that S‐phase progression varied widely between LmRad9+/− and LmHus1+/− cells. Therefore, we set out to investigate the effect of LmRad9 and LmHus1 deficiency on DNA synthesis. For this, we used fluorescence microscopy to analyze and measure EdU incorporation in exponentially growing LmRad9+/− and LmHus1+/− cells as compared with WT cells. We observed that the proportion of cells that incorporated EdU did not significantly vary between the three cell types (Supporting Information Figure S6B). However, the level of EdU incorporation in individual cells varied significantly, as shown in Fig. 4A and B and Supporting Information Figure S6C. LmRad9+/− cells incorporated significantly less EdU when compared with WT cells, indicating that reduction in LmRad9 causes a decrease in DNA synthesis. This observation is in agreement not only with the decrease in S‐phase progression rate observed in these cells, but also with their poor recovery from genotoxic stress (Fig. 3A and B). On the other hand, EdU incorporation was markedly increased in LmHus1+/− cells (Fig. 4A and B), indicating that these cells have an increased DNA synthesis rate, which correlates with their faster progression through the cell cycle and greater recovery after replication stress. Altogether, these data provide biological evidence that LmRad9 and LmHus1 can act by distinct mechanisms as cell cycle checkpoint factors, and that these divergent mechanisms affect DNA synthesis processes.

### LmRad9 and LmHus1 participate in the response to DSBs

**Figure 5:**
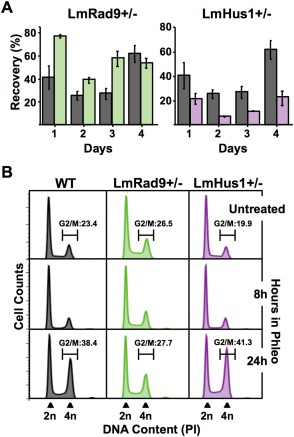
LmRad9 and LmHus1 have different roles in the response to DSBs. A. WT, LmRad9+/− and LmHus1+/− cells were treated with 10 μg/ml phleo for ∼15 h and seeded in drug‐free media at 10^5^ cells/ml; cell densities were assessed daily and recovery was calculated as a percentage of proliferation as compared with the nontreated cells; vertical lines on top of each bar indicate standard deviation. B. WT, LmRad9+/− and LmHus1+/− cells were incubated with 10 μg/ml of phleo for the indicated period of time prior to DNA content analysis by flow cytometry; data from 50000 events were analyzed in each time point; percentage showed for the untreated and 24 h‐treat cells indicate the proportion of cells at the G2/M transition; 2n and 4n indicate nonreplicated and replicated DNA, respectively.

Based on the results described above, we decided to investigate whether the distinct effects of LmRad9 and LmHus1 deficiencies are limited to the replication stress response or characterize a general response to DNA damage. Thus, we analyzed the recovery profile of LmRad9+/− and LmHus1+/− cells after exposure to the radiomimetic drug phleomycin (phleo), which causes a high frequency of DSBs in DNA (Moore, 1988). Contrary to what we observed in the response to replication stress by exposure to HU, CPT and MMS, phleo treatment caused LmRad9+/− cells to proliferate faster than WT cells in the first three days of growth following exposure (Fig. 5A). LmHus1+/− cells also presented a phleo recovery profile that was the opposite of that observed in response to HU, CPT or MMS treatments, and was again distinct from LmRad9+/− cells, with phleo‐treated LmHus1‐deficient cells exhibiting slower proliferation rate when compared with WT cells (Fig. 5A). These data indicate not only that both LmRad9 and LmHus1 are required for an appropriate response to DSBs, but that, as in the response to replication stress, the two proteins can play distinct roles in this pathway also.

We next asked if the marked differences in the proliferation rates between the two cell lines upon phleo exposure would correlate with alterations in the cell cycle profile. Thus, we again used flow cytometry analysis to assess DNA content in the LmRad9+/− and LmHus1+/− cells cultivated in the presence of phleo. As shown in Fig. 5B, we observed that WT cells presented a significant accumulation of cells in the G2/M phase after 24 h of incubation with phleo (∼ 1.5‐fold increase). In contrast, we did not observe any significant increase in the G2/M population of LmRad9+/− cells at the same time point. This finding agrees with the proliferation profile observed for these cells (Fig. 5A) and suggests that LmRad9 is required to arrest cells at the G2/M phase in response to DSBs. In agreement with lowered survival after phleo treatment, LmHus1+/− cells presented a noticeable increase (Fig. 5B) in the proportion of cells in the G2/M phase (∼2.0‐fold increase, which is greater than WT). This finding indicates not only that LmHus1 is required for proper cell cycle progression in the presence of DSBs, but also that its mode of action substantially differs from that of LmRad9 in these conditions.

### LmRad9 and LmHus1 participate in the DNA damage signaling upon genotoxic stress

**Figure 6:**
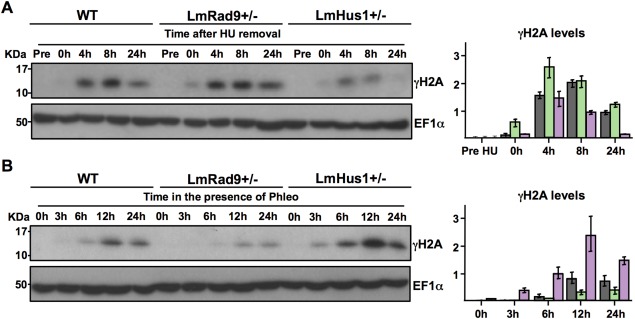
LmRad9 and LmHus1 deficiency has distinct effects on DNA damage persistence upon HU and phleo treatment. A. WT, LmHus1+/− and LmRad9+/− cells were incubated with 5mM HU for ∼10 h; after washing, cells were incubated in HU‐free medium and collected at the indicated time points; Pre indicate extracts prepared right before HU addition; extracts prepared in each time point were analyzed by western blot with anti‐γH2A antibody; EF1α was used as a loading control; graph at right shows quantification or γH2A signal as normalized with EF1α signal; vertical lines on top of each bar indicate standard deviation. B. WT, LmHus1+/− and LmRad9+/− cells were incubated with 5 μg/ml of phleo for the indicated periods of time; extracts prepared in each time point were analyzed by western blot with anti‐γH2A antibody; EF1α was used as a loading control; graph at right shows quantification or γH2A signal as normalized with EF1α signal; vertical lines on top of each bar indicate standard deviation.

To further investigate the phenotypes described above, we next analyzed the levels of γH2A in LmRad9+/− and LmHus1+/− cell lines exposed to HU or phleo. γH2A has been described as the *Trypanosoma brucei* equivalent of γH2AX (Glover and Horn, 2012), which is an early‐acting chromatin signal of DNA damage in other eukaryotes (Kinner *et al*., 2008). To validate the use of anti‐γH2A antiserum as a DSB marker in *L. major*, we performed western blot analysis of protein extracts from WT cells exposed to phleo for increasing periods of time. As presented in Supporting Information Figure S7, an increase in γH2A signal correlated with the increasing incubation with phleo, indicating that γH2A is generated in response to this treatment in *Leishmania* in the same way as seen in *T. brucei*. Therefore, we performed western blot analyses of protein extracts from WT, LmRad9+/− and LmHus1+/− cells after exposure to HU. As shown in Fig. 6A, LmRad9+/− cells presented an increase in γH2A levels between 0 and 4 h after the removal of HU. This finding suggests that the retention of LmRad9+/− cells in the S phase after HU arrest (Fig. 3B), is associated with DNA damage accumulation. On the other hand, LmHus1+/− cells presented a marked decrease in γH2A levels after 8 h from HU removal. This indicates that LmHus1+/− cells are defective on maintaining the DNA damage signaling resulting in a faster progression of these cells through G2/M transition (Fig. 3B).

We also analyzed the levels of H2A phosphorylation when cells were exposed to the DSB agent phleo. As shown in Fig. 6B, LmRad9+/− cells presented a significant reduction in γH2A levels even after prolonged incubation times with phleo (24 h). Strikingly, LmHus1+/− cells presented an increased level of H2A phosphorylation upon phleo incubation. This finding agrees with the recovery analyses presented in Fig. 5A and B, which suggest that LmRad9 is necessary for the DSBs signaling process that mediates halting of cells at the G2/M boundary after phleo treatment. Also, it indicates that DSB signaling is not only operative, but also increased in LmHus1+/− cells, causing cell proliferation to halt after phleo exposure. In addition, the divergent levels of γH2A in the LmHus1+/− and LmRad9+/− cells reinforce all previous assays that suggest the two factors can act independently.

### LmRad9 and LmHus1 form diverse complexes *in vivo*

**Figure 7:**
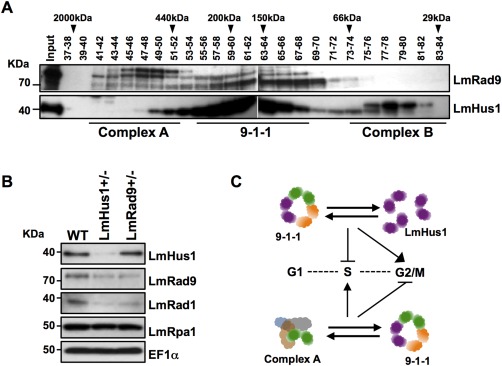
LmRad9 and LmHus1 can be found in distinct complexes in soluble cell extracts. A. Total cell extract from WT cells was subjected to fractionation in a Superdex‐200 column; the indicated fractions (numbers above each lane) were pooled and probed with anti‐LmRad9 and anti‐LmHus1 antibodies; arrowheads indicate peak elution fractions for calibration standards: dextran blue (2000 kDa), apoferritin (440 kDa), β‐amylase (200 kDa), alcohol dehydrogenase (150 kDa), bovine serum albumin (66 kDa), and carbonic anhydrase (29 kDa). B. Western blot analysis of total cell extract from WT, LmHus1+/− and LmRad9+/− cells; extracts were sequentially probed with anti‐LmHus1, anti‐LmRad9, anti‐LmRad1, and anti‐LmRpa1 antibodies; EIF1α was used as a loading control. C. Tentative model for the dynamics and function of the 9‐1‐1 subunits in *L. major*: in unperturbed *Leishmania* cells the subunits LmRad9, LmRad1, and LmHus1 are found in at least three configurations: complex A containing LmRad9, the 9‐1‐1 complex, and LmHus1 in its monomeric state; the stoichiometric balance among the different forms determines the roles of the three subunits; S‐phase progression after replication stress is positively modulated by the fine‐tuning between complex A and the 9‐1‐1 complex, and negatively modulated by the equilibrium between the 9‐1‐1 clamp and monomeric LmHus1; also, the G2/M transition upon DSBs is restricted by the interplay between complex A and the 9‐1‐1 complex and facilitated by the balance between the 9‐1‐1 clamp and free LmHus1.

To understand the divergent phenotypes observed upon LmRad9 and LmHus1 deficiency, we performed size‐exclusion chromatography with soluble cell lysates and investigated the elution pattern of LmRad9 and LmHus1. This analysis revealed that LmRad9 (predicted molecular mass ∼73 kDa) and LmHus1 (∼37 kDa) have a coincident elution peak (fractions 57–68) with an apparent molecular mass between 150 and 200 kDa (Fig. 7A). LmRad1 presented a similar pattern in gel filtration experiments with an elution peak between fractions 56–64 (Supporting Information Figure S8). The apparent molecular mass for the elution peak containing the three proteins is consistent with the size of a 9‐1‐1 complex containing LmRad9, LmRad1, and LmHus1 and is also consistent with the coIP experiment presented in Fig. 1. Intriguingly, we also observed that LmRad9 is contained in a complex with an apparent molecular mass of more than 440 kDa (hereafter named complex A), which does not seem to include LmHus1 (Fig. 7A) or LmRad1 (Supporting Information Figure S8). In addition, we observed that LmHus1 is also not only found in the 9‐1‐1 complex‐containing fractions, since we detected it in elution fractions corresponding with lower molecular weight (∼40 kDa, complex B), consistent with the predicted monomeric molecular mass of LmHus1. These findings perhaps explain the phenotypes reported above: if LmRad9 and LmHus1 are not solely present in the 9‐1‐1 complex, the differing responses to DNA damage in the LmRad9 or LmHus1 deficient cell lines can be rationalized.

We next analyzed the levels of the three subunits in LmRad9+/− and LmHus1+/− cell lines. As presented in Fig. 7B, LmHus1 depletion had the most prominent effect, since the levels of both LmRad9 and LmRad1 (in addition to LmHus1 itself) were markedly decreased in the LmHus1+/− cell line. In contrast, depletion of LmRad9 led to a substantial reduction in LmRad1 levels but did not affect the levels of LmHus1. The existence of some LmHus1 as a monomer may explain why its levels are not significantly reduced upon LmRad9 deficiency, whereas the association of LmRad9 in two complexes may mean it is unstable (and its levels reduce) upon LmHus1 deficiency. Altogether, the data presented suggest that these diverse complexes may constitute the molecular basis of the functional compartmentalization of LmRad9 and LmHus1 in the response to genotoxic stress in *Leishmania*.

## Discussion

In this report, we have extended the characterization of the putative 9‐1‐1 homolog complex of *L. major*. Our data not only qualifies the complex as a bona fide participant of *Leishmania* DNA metabolism, but also confirms the existence of operating 9‐1‐1 subunits in this early‐branching eukaryote. Some of the canonical features of the complex seem to be conserved in the protozoan, such as the recruitment of its subunits to chromatin in response to replication stress. Also, LmRad9 and LmHus1 are both required for telomere maintenance and the efficient cell response to genotoxic stress. Although the data indicate the participation of these proteins in similar pathways, the phenotypic analysis of the LmRad9 and LmHus1 deficient cell lines suggests a functional compartmentalization between these two subunits. In alignment with this, we observed that besides a complex bearing both LmRad9 and LmHus1, LmRad9 can form an alternative complex and LmHus1 can also be found in its monomeric form. Thus, it is possible that LmRad9 and LmHus1 have evolved to form a variety of complexes, which correlate with their functional compartmentalization and indicate a marked divergence of the 9‐1‐1 subunits homologs in this parasite.

Sequence divergence of the *L. major* 9‐1‐1 homolog subunits hampered initial identification of their coding sequences within the parasite genome. The use of conventional BLAST searches did not reveal a *Leishmania* Rad1 homolog. Identification of ORF LmjF.20.0390 as encoding LmRad1 was achieved only by probing the annotated *Leishmania* genome with the tertiary structure of the human Rad1, and using iterative PSI‐BLAST (MacNeill, 2014; Kelley *et al*., 2015). The biochemical characterization of the LmjF.20.0390 product reported here confirms it as the Rad1 homolog of *Leishmania* by demonstrating that, similar to LmRad9 and LmHus1, it is present in the 9‐1‐1 complex, as shown by the coIP and gel‐filtration experiments. Similarly to LmRad9 and LmHus1, LmRad1 also associates with chromatin in response to genotoxic stress. Nonetheless, a marked divergence of the *Leishmania* 9‐1‐1 clamp is indicated by structural modelling of its subunits, which showed that the predicted tertiary structure of the carboxy‐terminal regions of LmRad9 and LmRad1 are significantly diverged from the crystal structure available for homologs from other eukaryotes. The carboxyl‐domains of human Rad9 and Rad1 bear the interaction interface with Hus1 and Rad9, respectively (Dore *et al*., 2009). The predicted structural divergence of LmRad9 and LmRad1 does not seem to compromise either their association in the 9‐1‐1 complex or their ability to interact with the chromatin. However, this predicted structural changes might underlie the functional compartmentalization we observed for the LmRad9 and LmHus1 subunits, perhaps through providing a diverse platform of interaction with other proteins or regulatory factors.

In contrast with PCNA, the heterotrimeric nature of the eukaryotic 9‐1‐1 clamp adds complexity to its regulation and might expand its range of functions. In mammals and yeast, the functional diversification of the 9‐1‐1 clamp results from the appearance of Hus1 and Rad9 paralogs and isoforms (Lyndaker *et al*., 2013b). As an example, the formation of noncanonical complexes involving Rad9B, Rad1 and Hus1B has been suggested as an essential factor for proper completion of mammalian meiosis (Lyndaker *et al*., 2013b). It has also been reported that the human paralog Rad9B seems to be involved not only in the G1/S transition, but also in the response to nucleolar stress (Perez‐Castro and Freire, 2012). In *Schizosaccharomyces pombe*, functional diversification of the 9‐1‐1 complex has involved the generation of Rad9 isoforms through translation control, allowing one isoform to modulate mitotic commitment upon heat shock stress (Janes *et al*., 2012). Although we have not been able to identify paralogs of LmRad9, LmRad1, or LmHus1 in *Leishmania*, three sets of data indicate a functional diversification between the three subunits. First, phylogenetic analysis and the structural modelling of each subunit predict that they have diverged at different rates (Fig. 1A and Supporting Information Figure S1). Second, depletion of LmRad9 or LmHus1 had markedly different effects on the levels of the other subunits, which may account for the differences in the phenotypes we have observed in these cells (see below). Third, size exclusion chromatography indicates the presence of LmRad9 and LmHus1 outside the context of a canonical 9‐1‐1 homolog clamp. All these observations indicate that the 9‐1‐1 subunits of *Leishmania* provide functions that surpass the context of a canonical clamp, meaning that functional diversification of the 9‐1‐1 clamp has also occurred in this parasite, though without the generation of variant subunits.

Diminished levels of either LmRad9 or LmHus1 not only affected cell proliferation, but also interfered in telomere homeostasis, cell cycle progression, DNA synthesis, and responses to replication stress or drug‐induced DSBs. Despite this broad involvement in related aspects of genome maintenance, the detailed phenotypes of LmRad9 or LmHus1 deficient cell lines indicate that the modus operandi of these two 9‐1‐1 subunits differ considerably. In fact, for most of the phenotypes analyzed, LmRad9 and LmHus1 deficiencies gave rise to opposite phenotypes. While exposure to replication stress caused LmHus1 deficient cell lines to proliferate more quickly, LmRad9 deficiency led to a slower rate of cell proliferation. In line with this, LmHus1‐deficient cells displayed a deregulated transit through S phase, with an increased DNA synthesis rate and a faster G2/M transition. In contrast, LmRad9 deficiency led to a marked decrease in DNA synthesis, a consequent retention of cells in S phase and a slow progression through G2/M transition. The role of the 9‐1‐1 complex as a regulatory factor of DNA replication under genotoxic stress is widely documented. In mammals, lower levels of Rad9, Rad1 or Hus1 result in deregulated DNA synthesis after genotoxic stress (Roos‐Mattjus *et al*., 2003; Weiss *et al*., 2003; Bao *et al*., 2004). Moreover, recent data reveal that the interplay between 9‐1‐1 complex and DNA replication in *Saccharomyces cerevisiae* seems to occur through the physical interaction between Mec3 (Hus1) and Mcm10, a component of the pre‐replication complex (Alver *et al*., 2014). It remains to be determined whether the roles of LmRad9 or LmHus1 in *Leishmania* DNA synthesis involve a similar mechanism. It cannot be excluded that the altered DNA synthesis derived from LmRad9 or LmHus1 deficiencies could arise from a defective intra‐S checkpoint or altered levels of DNA damage repair. In fact, our data supports the last hypothesis, since the retention of LmRad9+/− cells on S phase and the faster progression of LmHus1+/− cells correlate with altered levels of γH2A following replication stress.

The functional compartmentalization between LmRad9 and LmHus1 seems to be extended to other aspect of genome maintenance in *L. major*. Phenotypic analysis of LmRad9 and LmHus1‐deficient cells also indicated differing contributions of LmRad9 and LmHus1 in the response to drug‐induced DSBs. LmHus1 deficiency resulted in a pronounced decrease in cell proliferation in response to phleomycin, while reduced levels of LmRad9 resulted in a defective proliferation arrest. In line with this, LmHus1 deficiency led to a detectable increase in H2A phosphorylation and an accumulation of cells in the G2/M phase after exposure to phleomycin, while LmRad9 deficiency resulted in the abrogation of H2A phosphorylation and failure to arrest cells in the G2/M phase. In other eukaryotes, Rad9 or Hus1 deficiencies interfere with the accumulation of DSB markers, such as Rad51 or γH2AX foci upon genotoxic stress, denoting that these proteins are required for proper repair of DSBs (Pandita *et al*., 2006; Lyndaker *et al*., 2013a). Also, Rad9 and Hus1 have been shown to be required in homologous recombination events in other eukaryotes (Ngo and Lydall, 2015). Our data indicate that LmRad9 and LmHus1 also participate in the *Leishmania* response to DSBs and it is therefore reasonable to speculate that LmRad9 and LmHus1 act in homologous recombination, which is a pivotal instrument for proficient gene amplification observed in *L. major* (Genois *et al*., 2015). Thus, it is conceivable that the 9‐1‐1 proteins have an unforeseen role in *Leishmania* gene amplification mechanisms. However, how the proteins might act is complicated by the observation that the mode of action of the two proteins in response to phleomycin treatment seems to differ. Thus, we cannot yet infer if LmRad9 and/or LmHus1 are involved in the actual processing of DSBs, like the yeast 9‐1‐1 clamp (Ngo and Lydall, 2015), or in the signaling events that culminate in the phosphorylation of the H2A histone. Nonetheless, the distinct contribution of LmRad9 and LmHus1 to the DSB response further demonstrates the functional compartmentalization of the two proteins, and examining their roles in gene amplification might reveal further features of how homologous recombination contributes to this process in *Leishmania*.

The available data on Rad9 and Hus1 from other eukaryotes suggests that the two proteins normally act together as components of the 9‐1‐1 complex, meaning that the functional compartmentalization of LmRad9 and LmHus1 in *Leishmania* is unprecedented. Size‐exclusion chromatography analyses of yeast cell extracts did not detect any of the 9‐1‐1 subunits in complexes other than the 9‐1‐1 clamp (Kondo *et al*., 1999; Caspari *et al*., 2000). Moreover, reduced levels of human Rad9 leads to uncontrolled DNA synthesis after genotoxic stress (Pandita *et al*., 2006), which is similar to the effect of Hus1 knockdown in mice cells (Weiss *et al*., 2003). Though a recent study presented evidence for an alternative 9‐1‐1 complex in human cells, this is formed by the interaction of each of the canonical 9‐1‐1 clamp subunits with the Rad9B paralog after human cells are treated with actinomycin D (Perez‐Castro and Freire, 2012). Nonetheless, analysis of mammalian cell extracts indicates that Rad1 can be found as a monomer (Burtelow *et al*., 2001), which correlates with a possible Rad9 or Hus1‐independent functions in meiotic cells (Lyndaker *et al*., 2013a).

Despite the novelty of the observations that we make here, it is noteworthy that most studies assume that the effect of the individual abrogation of Rad9, Rad1 or Hus1 expression is a consequence of the disruption of the entire 9‐1‐1 complex, without taking into account the possible existence of alternative complexes. Indeed, the dissection of functional compartmentalization of individual subunits is problematic due to dual functions within and outwith the 9‐1‐1 complex. Thus, it is possible that the phenotypes that we observe for LmRad9 and LmHus1 are detectable because of pronounced, lineage‐specific roles for the subunits outwith the *Leishmania* 9‐1‐1 complex, and such roles and interactions are present in other eukaryotes but have so far escaped detection. In this regard, it may be valuable to test 9‐1‐1 function in other kinetoplastids. Irrespective of the above evolutionary implications, the present study provides substantial support for the hypothesis that the global role of LmRad9 and LmHus1 results from their distribution between at least three arrangements: the canonical 9‐1‐1 complex, a distinct complex (“A”) bearing LmRad9 (and lacking LmHus1 and LmRad1) and monomeric LmHus1, as shown in the tentative model presented in Fig. 7C. In this model, the distinct roles of LmRad9 and LmHus1 in S phase or G2/M transition are most simply explained by functions provided by LmRad9 in complex A or LmHus1 function in its monomeric state. However, understanding the stoichiometric balance between these activities and the dynamics of interaction between these subunits is crucial to dissect the roles the proteins play, which appear to be essential, since null mutants could not be generated. In this context, a more complicated explanation for the opposite phenotypes observed upon LmRad9 and LmHus1 deficiencies might reside not in loss of activities provided by the proteins when operating outwith the 9‐1‐1 complex, but result from disturbance in the balances between complex A and 9‐1‐1, and the 9‐1‐1 clamp and the monomeric pool of LmHus1. Addressing all these questions will require analysis of the specific roles of complex A or monomeric LmHus1: for instance, what factors does LmRad9 interact with, and what activities might LmHus1 harbour in isolation? Also, what factors determine the recruitment of each subunit to the different complexes? Our gel‐filtration analysis indicates that the slower migrating forms of LmRad9 are predominant in complex A, which suggests that LmRad9 distribution between complexes may involve posttranslational modifications. In fact, the phosphorylation of Hus1 was shown in other eukaryotes (Caspari *et al*., 2000) and the carboxyl domain of Rad9 is found highly phosphorylated both constitutively and as a response to genotoxic stress in yeast and mammals (Roos‐Mattjus *et al*., 2003; Furuya *et al*., 2010).

In summary, our data indicate that the *Leishmania* 9‐1‐1 homolog subunits play a more widespread role in DNA metabolism than might have been anticipated. The separation of function between LmRad9 and LmHus1 seems to be based on the formation of at least one noncanonical LmRad9‐containing complex, and may reveal novel activities of monomeric Hus1. Further studies are required to dissect the activities of LmRad9 and LmHus1 and to evaluate their participation in key events that shape the biology of *Leishmania* and related protozoa.

## Experimental procedures

### Parasite culture

*L. major* LT252 (MHOM/IR/1983/IR) and transfected cell lines derived from this strain were cultured as promastigotes in M199 medium containing 10% heat‐inactivated fetal bovine serum at 26°C. The heterozygous or episome‐bearing cell lines were generated using the transfection protocol previously described (Kapler *et al*., 1990). The cell lines LmRad9+/−, LmRad9+/−/+, OE^Rad9^ and LmHus1+/− were cultivated in the presence of 16 μg/ml hygromycin, 16 μg/ml hygromycin plus 8μg/ml G418, 8μg/ml G418 plus 40 μg/ml nourseothricin; respectively.

### Antibodies and western blotting analysis

Rabbit anti‐LmHus1 and anti‐LmRad9 antibodies were previously described (Nunes *et al*., 2011; Damasceno *et al*., 2013). The rabbit anti‐LmRpa1 antibody was raised against recombinant 6xHis‐LmRpa1expressed in *E. coli* BL21 cells, from the pET28a‐LmjF.28.1820 construct (Proteimax; www.proteimaxnet.com.br); serum was further affinity‐purified using the recombinant protein as bait. The chicken anti‐LmRad1 antibody was raised against recombinant 6xHis‐LmRad1expressed in *E. coli* BL21 cells from the pET28aLmjF.20.0390 construct, and purified by ammonium sulfate precipitation and ion exchange chromatography. The mouse anti‐LmRad1 serum was also raised against recombinant 6xHis‐LmRad1. The anti‐γH2A antibody was generated by immunizing rabbit with the phospho‐peptide KHAKA[pT]PSV (Thermo Fisher Scientific; Waltham, MA, USA); serum was further affinity‐purified using the corresponding peptide as bait. The commercial antibodies used were as follows: anti‐Myc and anti‐EF1α (Merck Millipore; Billerica, MA, USA); anti‐H2A (Santa Cruz; Dallas, TX, USA); anti‐HA (Sigma; St. Louis, MO, USA). For western blotting analysis, proteins were resolved in SDS‐PAGE, transferred to PVDF membrane and analyzed with the indicated antibodies. Bands were detected with ECL Prime Western Blotting Detection Reagent (GE Life Sciences; Pittsburgh, PA, USA) and visualized with Hyperfilm ECL (GE Life Sciences) or ImageQuant LAS 4000 (GE Life Sciences). For data in Figs 6 and 1C, western blotting analysis was performed with SuperSignal® Western Blot Enhancer (Thermo Scientific), following manufacturer instructions, and signal detection was performed with ECL Prime Western Blotting Detection Reagent.

### *In vitro* translation and pull down assay

LmjF.20.0390 ORF was cloned into pTCFE1‐NHA vector. The resulting pTCFE1‐NHA‐LmRad1 construct was used with the 1‐Step Human‐Coupled IVT Kit (Thermo Scientific), according to manufacturer instructions, to generate the HA‐LmRad1 fusion. HA‐LmRad1 was immobilized with protein A‐agarose beads coupled with anti‐HA antibody. Total cell extracts from *L. major* was prepared by suspending cells in pull down buffer (50 mM Tris, pH 7.5; 130 mM KCl; 10% Glycerol; 0.05% NP40; 10 mM Na_3_VO_4_ and 1X Roche protease inhibitor cocktail). After brief sonication, extracts were clarified by centrifugation (15 min; 15,000×*g*; 4°C) and incubated with immobilized HA‐LmRad1 for ∼2 h at 4°C under agitation. Beads were washed with pull down buffer and analyzed by western blotting.

### Co‐immunoprecipitation

For co‐immunoprecipitation (coIP) ∼10^9^ cells were harvested and lyzed in ice cold Buffer A (50mM Tris pH 9.0; 400mM KCl; 1% NP40; 10% Glycerol; 10mM EDTA; 10mM EGTA; 5mM DTT; 5mM Na_3_VO_4_; 5mM β‐glycerophosphate disodium; 5× Roche protease inhibitor cocktail). After clarification with centrifugation (30 min; 20,000×*g*; 4°C), extracts were incubated for ∼4 h at 4°C with Dynabeads M‐280 Sheep Anti‐Mouse IgG beads (Thermo Fisher Scientific) previously coupled with either mouse anti‐LmRad1 serum or mouse anti‐Myc monoclonal antibody. Beads were collected with a magnetic rack and washed 3× in Buffer A. CoIP products were suspended in Laemmli and analyzed by western blotting.

### Cell fractionation

Soluble and chromatin bound proteins were fractionated as described before (Godoy *et al*., 2009). Briefly, ∼5 × 10^7^ cells were harvested, washed with 1xPBS and incubated with extraction buffer (10 mM Tris‐HCl pH 9.0, 100 mM NaCl, 0.1% Triton X‐100, 300 mM sucrose, 3 mM MgCl_2_, 5 mM Na_3_VO_4_, 5mM β‐glycerophosphate disodium; 3X Roche protease inhibitor cocktail) for 10 min on ice. The suspension was centrifuged (5 min; 3000×*g*; 4°C) and the supernatant was saved as soluble fraction (Soluble I). The precipitated insoluble material was treated with extraction buffer again, centrifuged as before and the supernatant was saved as soluble fraction (Soluble II). The pellets were treated with DNaseI Amplification Grade (Invitrogen; Waltham, MA, USA) (10 units for 5 × 10^7^ cells) for 20 min at room temperature. The sample was centrifuged (5 min; 5000×*g*; 4°C), and the supernatant was saved as DNaseI released fraction (Chromatin).

### Size‐exclusion chromatography

Soluble extracts were prepared from ∼5 × 10^9^ exponentially growing cells suspended in lysis buffer (50 mM HEPES, pH 7.5; 150 mM KCl; 10% Glycerol; 0.1% NP40; 5 mM Na_3_VO_4_; 5 mM β‐glycerophosphate disodium; 5 mM NaF; 3X Roche protease inhibitor cocktail). Lysis was performed on ice for 30 min. Extracts were clarified by ultracentrifugation (1 h; 100,000×*g*; 4°C). Supernatant was applied to a Superdex 200 column (HiLoad^TM^ 16/60; GE Life Sciences) previously equilibrated with lysis buffer and connected to ÄKTA purifier system (GE Life Sciences). Lysate was resolved with a flow rate of 750 μl per minute and 1 ml fractions were collected.

### Flow cytometry analysis

Cells were harvested, washed with 1X PBS and fixed in 30% PBS/70% methanol overnight at 4°C. Fixed cells were washed with 1X PBS and stained in 1X PBS containing Propidium Iodide (50 µg/ml) and RNase A (100 µg/ml) at 37°C for 20 min. Flow cytometry data was collected using a BD FACSCanto flow cytometer (for fig. 3) and BD FACSCalibur (for fig. 5). Data were analyzed using the FlowJo software.

### Southern blotting analysis

Extraction of genomic DNA was performed as described before (Damasceno *et al*., 2013). DNA was treated with the indicated restriction endonucleases and digestion products were resolved by gel electrophoresis (0.6% agarose; 1X TAE; at 20V; for 16 h). DNA was transferred to Hybond‐N+ membranes (GE Life Sciences) and analyzed with indicated probes. Hybridization was carried at 65°C using AlkPhos Direct Labelling and Detection System with CDP‐Star (GE Life Sciences).

### EdU incorporation and quantification

Exponentially growing cells were incubated with 10 µM of EdU (Click‐iT EdU Image Kit; Thermo Scientific) for 2 h. Cells were fixed with 3.7% Formaldehyde for 15 min and then adhered into poly‐L‐lysine coated slides. Cells were permeabilized with 0.5% TritonX100 for 20 min, washed three times with PBS‐3% BSA and then incubated with the Click‐iT reaction cocktail for 30 min at room temperature. DNA was stained with Hoechst 33342. Images were acquired with a LSM 780 Axio Observer microscope (Zeiss; Oberkochen, Germany). Quantification of EdU fluorescence intensity was performed with ImageJ software.

## Author contributions

[JDD] planned and performed the experiments and wrote the manuscript; [RO] did the Southern blot presented on supplementary Supporting Information Figure S3, the PCR on Fig. 2 and the EdU incorporation analysis in Fig. 4; [EVS] raised the anti‐LmRad1 antibody and performed the western blot analyses in Fig. 1; [AS] assisted in the size‐exclusion chromatography analysis in Figs 1 and 6; [RM] assisted in planning the experiments and critically reviewed the manuscript; [LROT] planned the experiments and wrote the manuscript.

## Supporting information

Supporting Information.

## References

[mmi13441-bib-0001] Ahmed, S. , and Hodgkin, J. (2000) MRT‐2 checkpoint protein is required for germline immortality and telomere replication in *C. elegans* . Nature 403: 159–164. 10646593 10.1038/35003120

[mmi13441-bib-0002] Alver, R.C. , Zhang, T. , Josephrajan, A. , Fultz, B.L. , Hendrix, C.J. , Das‐Bradoo, S. , and Bielinsky, A.K. (2014) The N‐terminus of Mcm10 is important for interaction with the 9‐1‐1 clamp and in resistance to DNA damage. Nucleic Acids Res 42: 8389–8404. 24972833 10.1093/nar/gku479 PMC4117747

[mmi13441-bib-0003] Bao, S. , Lu, T. , Wang, X. , Zheng, H. , Wang, L.E. , Wei, Q. , *et al* (2004) Disruption of the Rad9/Rad1/Hus1 (9‐1‐1) complex leads to checkpoint signaling and replication defects. Oncogene 23: 5586–5593. 15184880 10.1038/sj.onc.1207753

[mmi13441-bib-0004] Bermudez, V.P. , Lindsey‐Boltz, L.A. , Cesare, A.J. , Maniwa, Y. , Griffith, J.D. , Hurwitz, J. , and Sancar, A. (2003) Loading of the human 9‐1‐1 checkpoint complex onto DNA by the checkpoint clamp loader hRad17‐replication factor C complex in vitro. Proc Natl Acad SciU S A 100: 1633–1638. 10.1073/pnas.0437927100 PMC149884 12578958

[mmi13441-bib-0005] Beverley, S.M. (1991) Gene amplification in *Leishmania* . Annu Rev Microbiol 45: 417–444. 1741620 10.1146/annurev.mi.45.100191.002221

[mmi13441-bib-0006] Burtelow, M.A. , Roos‐Mattjus, P.M. , Rauen, M. , Babendure, J.R. , and Karnitz, L.M. (2001) Reconstitution and molecular analysis of the hRad9‐hHus1‐hRad1 (9‐1‐1) DNA damage responsive checkpoint complex. J Biol Chem 276: 25903–25909. 11340080 10.1074/jbc.M102946200

[mmi13441-bib-0007] Caspari, T. , Dahlen, M. , Kanter‐Smoler, G. , Lindsay, H.D. , Hofmann, K. , Papadimitriou, K. , *et al* (2000) Characterization of *Schizosaccharomyces pombe* Hus1: A PCNA‐related protein that associates with Rad1 and Rad9. Mol Cell Biol 20: 1254–1262. 10648611 10.1128/mcb.20.4.1254-1262.2000 PMC85258

[mmi13441-bib-0008] Damasceno, J.D. , Nunes, V.S. , and Tosi, L.R. (2013) LmHus1 is required for the DNA damage response in *Leishmania major* and forms a complex with an unusual Rad9 homologue. Mol Microbiol 90: 1074–1087. 24118609 10.1111/mmi.12418

[mmi13441-bib-0009] Dore, A.S. , Kilkenny, M.L. , Rzechorzek, N.J. , and Pearl, L.H. (2009) Crystal structure of the rad9‐rad1‐hus1 DNA damage checkpoint complex–implications for clamp loading and regulation. Mol Cell 34: 735–745. 19446481 10.1016/j.molcel.2009.04.027

[mmi13441-bib-0010] Francia, S. , Weiss, R.S. , Hande, M.P. , Freire, R. , and D'adda di Fagagna, F. (2006) Telomere and telomerase modulation by the mammalian Rad9/Rad1/Hus1 DNA‐damage‐checkpoint complex. Curr Biol 16: 1551–1558. 16890531 10.1016/j.cub.2006.06.066

[mmi13441-bib-0011] Furuya, K. , Miyabe, I. , Tsutsui, Y. , Paderi, F. , Kakusho, N. , Masai, H. , *et al* (2010) DDK phosphorylates checkpoint clamp component Rad9 and promotes its release from damage chromatin. Mol Cell 40: 606–628. 21095590 10.1016/j.molcel.2010.10.026

[mmi13441-bib-0012] Genois, M.M. , Plourde, M. , Ethier, C. , Roy, G. , Poirier, G.G. , Ouellette, M. , and Masson, J.Y. (2015) Roles of Rad51 paralogs for promoting homologous recombination in *Leishmania* infantum. Nucleic Acids Res 43: 2701–2715. 25712090 10.1093/nar/gkv118 PMC4357719

[mmi13441-bib-0013] Glover, L. , and Horn, D. (2012) Trypanosomal histone gammaH2A and the DNA damage response. Mol Biochem Parasitol 183: 78–83. 22353557 10.1016/j.molbiopara.2012.01.008 PMC3334830

[mmi13441-bib-0014] Godoy, P.D. , Nogueira‐Junior, L.A. , Paes, L.S. , Cornejo, A. , Martins, R.M. , Silber, A.M. , *et al* (2009) Trypanosome prereplication machinery contains a single functional orc1/cdc6 protein, which is typical of archaea. Eukaryot Cell 8: 1592–1603. 19717742 10.1128/EC.00161-09 PMC2756867

[mmi13441-bib-0015] Hofmann, E.R. , Milstein, S. , Boulton, S.J. , Ye, M. , Hofmann, J.J. , Stergiou, L. , *et al* (2002) Caenorhabditis elegans HUS‐1 is a DNA damage checkpoint protein required for genome stability and EGL‐1‐mediated apoptosis. Curr Biol 12: 1908–1918. 12445383 10.1016/s0960-9822(02)01262-9

[mmi13441-bib-0016] Hwang, B.J. , Jin, J. , Gunther, R. , Madabushi, A. , Shi, G. , Wilson, G.M. , and Lu, A.L. (2015) Association of the Rad9‐Rad1‐Hus1 checkpoint clamp with MYH DNA glycosylase and DNA. DNA Repair 31: 80–90. 26021743 10.1016/j.dnarep.2015.05.004 PMC4458174

[mmi13441-bib-0017] Janes, S. , Schmidt, U. , Ashour Garrido, K. , Ney, N. , Concilio, S. , Zekri, M. , and Caspari, T. (2012) Heat induction of a novel Rad9 variant from a cryptic translation initiation site reduces mitotic commitment. J Cell Sci 125: 4487–4497. 22797921 10.1242/jcs.104075

[mmi13441-bib-0018] Kapler, G.M. , Coburn, C.M. , and Beverley, S.M. (1990) Stable transfection of the human parasite *Leishmania major* delineates a 30‐kilobase region sufficient for extrachromosomal replication and expression. Mol Cell Biol 10: 1084–1094. 2304458 10.1128/mcb.10.3.1084-1094.1990 PMC360971

[mmi13441-bib-0019] Kelley, L.A. , Mezulis, S. , Yates, C.M. , Wass, M.N. , and Sternberg, M.J. (2015) The Phyre2 web portal for protein modeling, prediction and analysis. Nat Protoc 10: 845–858. 25950237 10.1038/nprot.2015.053 PMC5298202

[mmi13441-bib-0020] Kinner, A. , Wu, W. , Staudt, C. , and Iliakis, G. (2008) Gamma‐H2AX in recognition and signaling of DNA double‐strand breaks in the context of chromatin. Nucleic Acids Res 36: 5678–5694. 18772227 10.1093/nar/gkn550 PMC2553572

[mmi13441-bib-0021] Kondo, T. , Matsumoto, K. , and Sugimoto, K. (1999) Role of a complex containing Rad17, Mec3, and Ddc1 in the yeast DNA damage checkpoint pathway. Mol Cell Biol 19: 1136–1143. 9891048 10.1128/mcb.19.2.1136 PMC116043

[mmi13441-bib-0022] Lyndaker, A.M. , Lim, P.X. , Mleczko, J.M. , Diggins, C.E. , Holloway, J.K. , Holmes, R.J. , *et al* (2013a) Conditional inactivation of the DNA damage response gene Hus1 in mouse testis reveals separable roles for components of the RAD9‐RAD1‐HUS1 complex in meiotic chromosome maintenance. PLoS Genet 9: e1003320. 23468651 10.1371/journal.pgen.1003320 PMC3585019

[mmi13441-bib-0023] Lyndaker, A.M. , Vasileva, A. , Wolgemuth, D.J. , Weiss, R.S. , and Lieberman, H.B. (2013b) Clamping down on mammalian meiosis. Cell Cycle 12: 3135–3145. 24013428 10.4161/cc.26061 PMC3865008

[mmi13441-bib-0024] MacNeill, S.A. (2014) Identification of a candidate rad1 subunit for the kinetoplastid 9‐1‐1 (rad9‐hus1‐rad1) complex. Biol (Basel) 3: 922–927. 10.3390/biology3040922 PMC4280517 25534152

[mmi13441-bib-0025] Medhurst, A.L. , Warmerdam, D.O. , Akerman, I. , Verwayen, E.H. , Kanaar, R. , Smits, V.A. , and Lakin, N.D. (2008) ATR and Rad17 collaborate in modulating Rad9 localisation at sites of DNA damage. J Cell Sci 121: 3933–3940. 19020305 10.1242/jcs.033688

[mmi13441-bib-0026] Moore, C.W. (1988) Internucleosomal cleavage and chromosomal degradation by bleomycin and phleomycin in yeast. Cancer Res 48: 6837–6843. 2460230

[mmi13441-bib-0027] Murta, S.M.F. , Vickers, T.J. , Scott, D.A. , and Beverley, S.M. (2009) Methylene tetrahydrofolate dehydrogenase/cyclohydrolase and the synthesis of 10‐CHO‐THF are essential in *Leishmania major* . Mol Microbiol 71: 1386–1401. 19183277 10.1111/j.1365-2958.2009.06610.x PMC2692627

[mmi13441-bib-0028] Nakamura, T.M. , Moser, B.A. , and Russel, P. (2002) Telomere binding of checkpoint sensor and DNA repair proteins contributes to maintenance of functional fission yeast telomeres. Genetics 161: 1437–1453. 12196391 10.1093/genetics/161.4.1437 PMC1462227

[mmi13441-bib-0029] Ngo, G.H. , and Lydall, D. (2015) The 9‐1‐1 checkpoint clamp coordinates resection at DNA double strand breaks. Nucleic Acids Res 43: 5017–5032. 25925573 10.1093/nar/gkv409 PMC4446447

[mmi13441-bib-0030] Nunes, V.S. , Damasceno, J.D. , Freire, R. , and Tosi, L.R. (2011) The Hus1 homologue of *Leishmania major* encodes a nuclear protein that participates in DNA damage response. Mol Biochem Parasitol 177: 65–69. 21291918 10.1016/j.molbiopara.2011.01.011

[mmi13441-bib-0031] Pandita, R.K. , Sharma, G.G. , Laszlo, A. , Hopkins, K.M. , Davey, S. , Chakhparonian, M. , *et al* (2006) Mammalian Rad9 plays a role in telomere stability, S‐ and G2‐phase‐specific cell survival, and homologous recombinational repair. Mol Cell Biol 26: 1850–1864. 16479004 10.1128/MCB.26.5.1850-1864.2006 PMC1430264

[mmi13441-bib-0032] Perez‐Castro, A.J. , and Freire, R. (2012) Rad9B responds to nucleolar stress through ATR and JNK signalling, and delays the G1‐S transition. J Cell Sci 125: 1152–1164. 22399810 10.1242/jcs.091124

[mmi13441-bib-0033] Rogers, M.B. , Hilley, J.D. , Dickens, N.J. , Wilkes, J. , Bates, P.A. , Depledge, D.P. , *et al* (2011) Chromosome and gene copy number variation allow major structural change between species and strains of *Leishmania* . Genome Res 21: 2129–2142. 22038252 10.1101/gr.122945.111 PMC3227102

[mmi13441-bib-0034] Roos‐Mattjus, P. , Hopkins, K.M. , Oestreich, A.J. , Vroman, B.T. , Johnson, K.L. , Naylor, S. , *et al* (2003) Phosphorylation of human Rad9 is required for genotoxin‐activated checkpoint signaling. J Biol Chem 278: 24428–24437. 12709442 10.1074/jbc.M301544200

[mmi13441-bib-0035] Ubeda, J.M. , Raymond, F. , Mukherjee, A. , Plourde, M. , Gingras, H. , Roy, G. , *et al* (2014) Genome‐wide stochastic adaptive DNA amplification at direct and inverted DNA repeats in the parasite *Leishmania* . PLoS Biol 12: e1001868. 24844805 10.1371/journal.pbio.1001868 PMC4028189

[mmi13441-bib-0036] Weiss, R.S. , Leder, P. , and Vaziri, C. (2003) Critical role for mouse Hus1 in an S‐phase DNA damage cell cycle checkpoint. Mol Cell Biol 23: 791–803. 12529385 10.1128/MCB.23.3.791-803.2003 PMC140711

[mmi13441-bib-0037] Xu, X. , Vaithiyalingam, S. , Glick, G.G. , Mordes, D.A. , Chazin, W.J , and Cortez, D. (2008) The basic cleft of RPA70N binds multiple checkpoint proteins, including RAD9, to regulate ATR signaling. Mol Cell Biol 28: 7345–7353. 18936170 10.1128/MCB.01079-08 PMC2593429

[mmi13441-bib-0038] Zou, L. , and Elledge, S.J. (2003) Sensing DNA damage through ATRIP recognition of RPA‐ssDNA complexes. Science 300: 1542–1548. 12791985 10.1126/science.1083430

[mmi13441-bib-0039] Zou, L. , Liu, D. , and Elledge, S.J. (2003) Replication protein a‐mediated recruitment and activation of Rad17 complexes. Proc Natl Acad Sci U S A 100: 13827–13832. 14605214 10.1073/pnas.2336100100 PMC283506

