# Supplementary material for: Functional compartmentalization of Rad9 and Hus1 reveals diverse assembly of the 9‐1‐1 complex components during the DNA damage response in Leishmania
Source: Mol Microbiol. 2016 Jul 19;101(6):1054–68. doi: 10.1111/mmi.13441 (PMC5453112; doi:10.1111/mmi.13441)
Supplement: Supplementary file 1 — Supporting Information. [file MMI-101-1054-s001.pdf]

SUPPLEMENTARY INFORMATION

Functional compartmentalization of Rad9 and Hus1 reveals diverse assembly of the 9-1-1 complex components during the DNA damage response in *Leishmania*

Jeziel D. Damasceno[1], Ricardo Obonaga[1], Elaine V. Santos[1], Alan Scott[2], Richard McCulloch[2] and Luiz R. O. Tosi[1,*]

[1] Department of Cell and Molecular Biology, Ribeirão Preto Medical School, University of São Paulo, Ribeirão Preto, SP, 14049-900, Brazil

[2] The Wellcome Trust Centre for Molecular Parasitology, Institute of Infection, Immunity and Inflammation, University of Glasgow; 120 University Place, Glasgow, G128TA, United Kingdom

•To whom correspondence should be addressed. Tel: 55 16 36023117; Fax: 55 16 36020728; Email: luiztosi@fmrp.usp.br

This PDF file includes:

Supplementary Figure 1
Supplementary Figure 2
Supplementary Figure 3
Supplementary Figure 4
Supplementary Figure 5
Supplementary Figure 6

Supplementary Figure 1

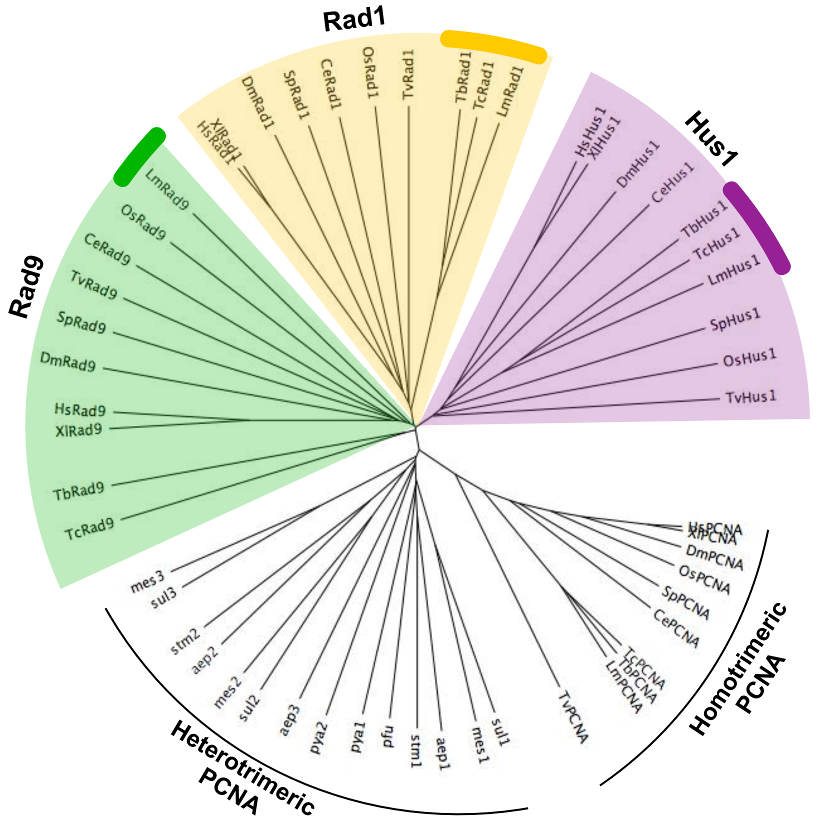

Figure S1. LmRad9, LmRad1 and LmHus1 are both phylogenetically and structurally related to 9-1-1 complex. Radial phylogram representation of sequence alignment of 9-1-1 and PCNA subunits from different eukaryotes and archea; the 9-1-1 subunits branches are colored and LmRad9, LmRad1 and LmHus1 homologues are highlighted in their respective groups; this analysis was based on previous work (Dore et al., 2009 and Damasceno et al., 2013); sequence alignment was generated with ClustalW multiple alignment tool (http://www.ebi.ac.uk/Tools/msa/clustalw2/) and used to build the phylogram on Dendroscope (http://ab.inf.uni-tuebingen.de/software/dendroscope/);

Supplementary Figure 2

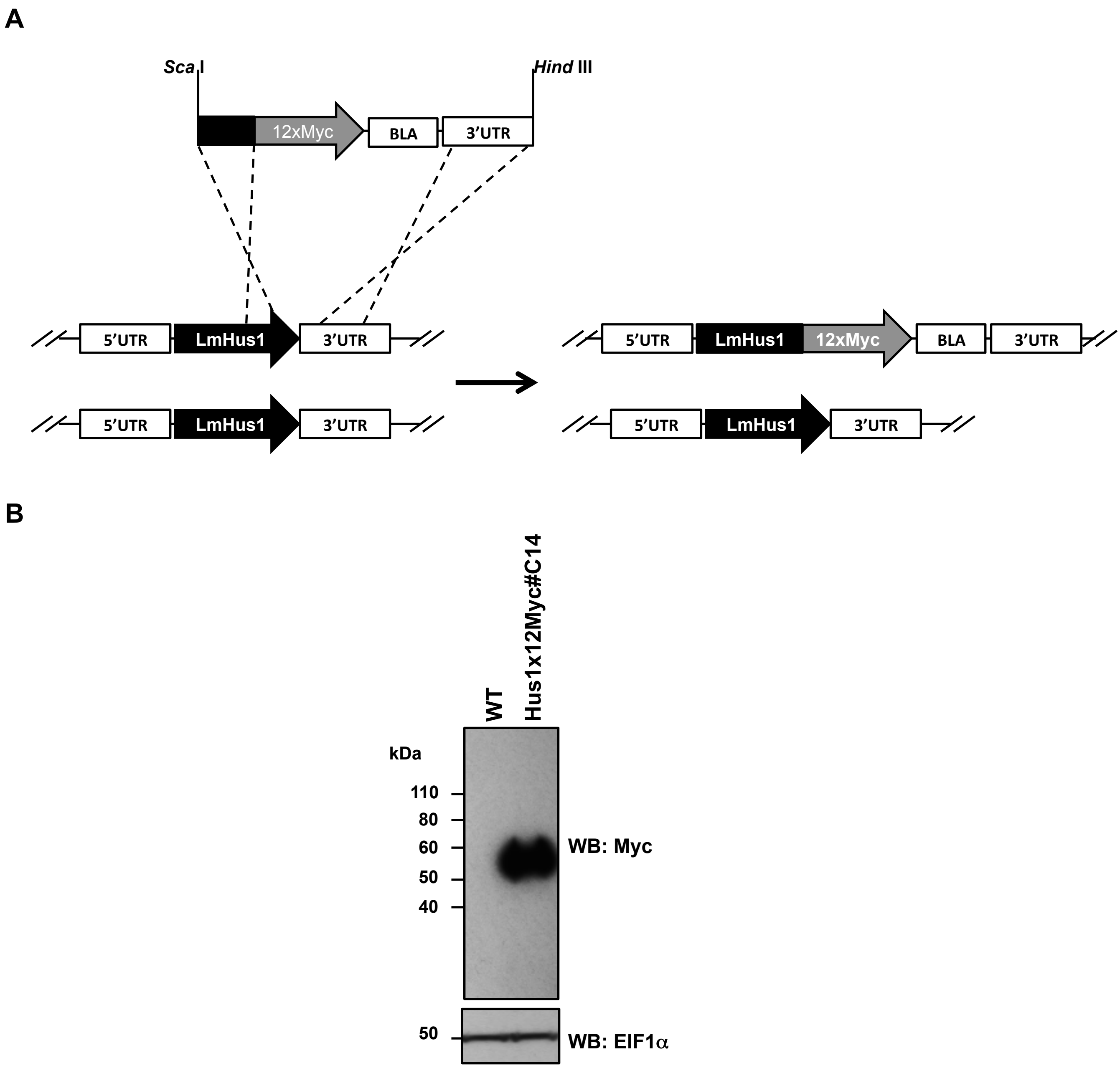

Figure S2. Detection of LmHus1-12xMyc expressed from the endogenous LmHus1 locus. A) A PCR amplified fragment from the 3' portion of LmHus1 ORF, lacking the stop codon, was cloned in frame with a 12xMyc sequence into the pNAT^{12MYC} vector (http://blogs.lshtm.ac.uk/alsfordlab/); the 3'UTR was also amplified and cloned downstream to the Blasticidin resistance cassette (BLA) coding sequence; the final construct was digested with *Sca*I and *Hind*III restriction enzymes and the linear fragment represented at the upper position was transfected into WT cells in order to replace one of the endogenous copy of LmHus1. **B)** Western blot analysis using anti-cMyc antibody of total protein lysate from WT and LmHus1-12xMyc expressing cells; the same membrane was also probed with anti- EF1α antibody to serve as a loading control.

Supplementary Figure 3

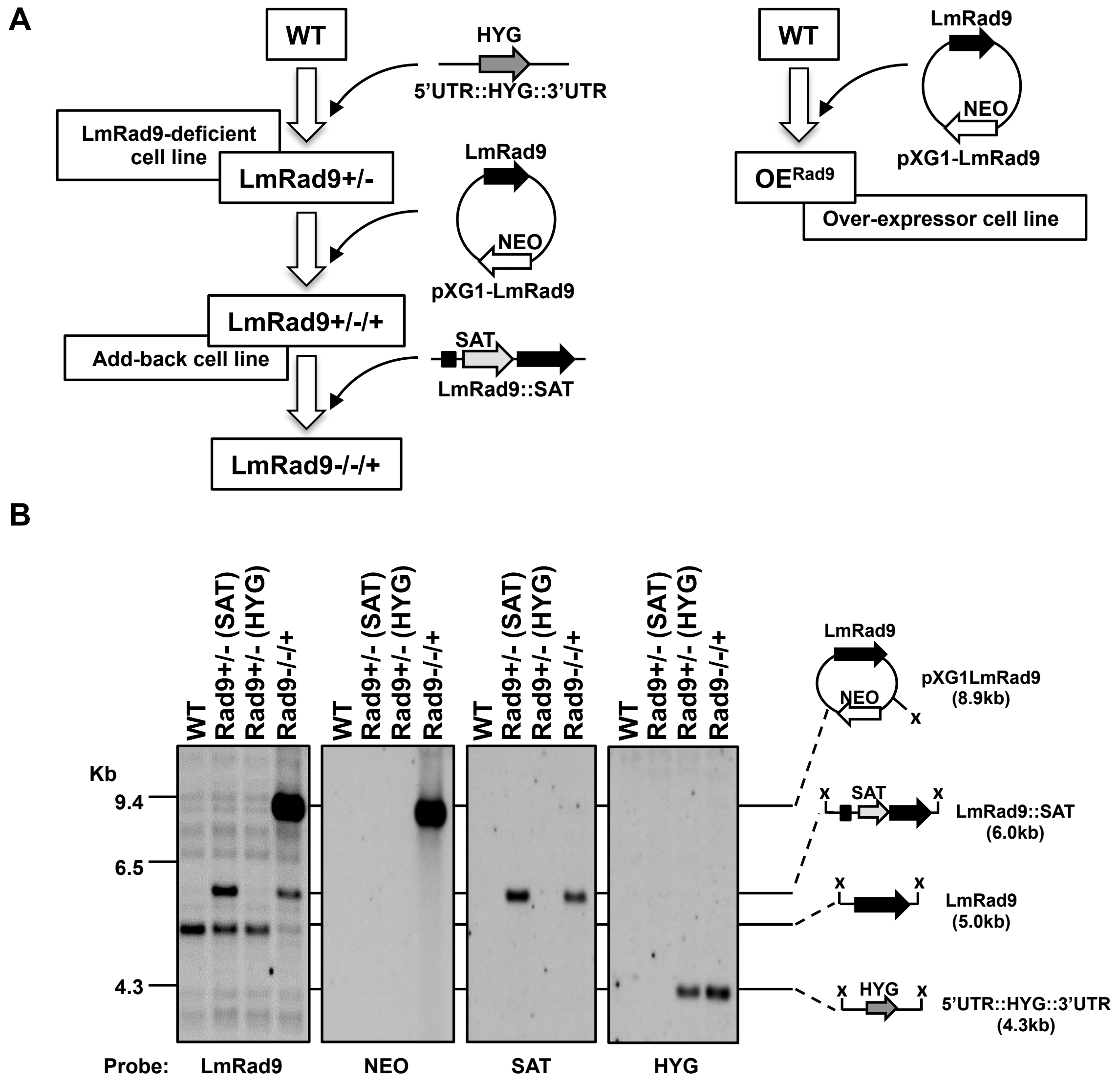

Figure S3. Characterization of LmRad9 mutant cell lines. A) Fluxogram of the strategy to generate LmRad9 mutant cells; to the left, the first LmRad9 allele was replaced by the hygromycin-resistance cassette (HYG) generating the LmRad9-deficient cell line (LmRad9+/-); the construct pXG1-LmRad9 was then transfected into LmRad9+/- to obtain the add-back cell line (LmRad9+/-/+); the remaining copy of LmRad9 was replaced by a non-functional copy of the gene disrupted with the nourseothricin-resistance cassette (SAT) generating the LmRad9-/-/+ cell line; to the right, the generation of an over-expressor cell line (OE^{Rad9}), in which both genomic copies of LmRad9 are intact and carries pXG1-LmRad9. **B)** Southern blot analysis of genomic DNA digested with restriction enzyme *Xho*I; DNA was analysed with the indicated probes bellow each panel; LmRad9+/-(SAT): cell line in which one allele of LmRad9 was disrupted by the SAT cassette; LmRad9+/-(HYG): cell line in which one allele of LmRad9 was replaced by a HYG cassette; at the right, schematic representation (not in scale) of the targeted disruption of the LmRad9 chromosomal and episome locus as predicted for the each cell line analysed; *x* indicates the approximated location of *Xho*I restriction sites; the numbers below each construct indicate the expected fragment size (in kb) of each construct after digestion with *Xho*I.

Supplementary Figure 4

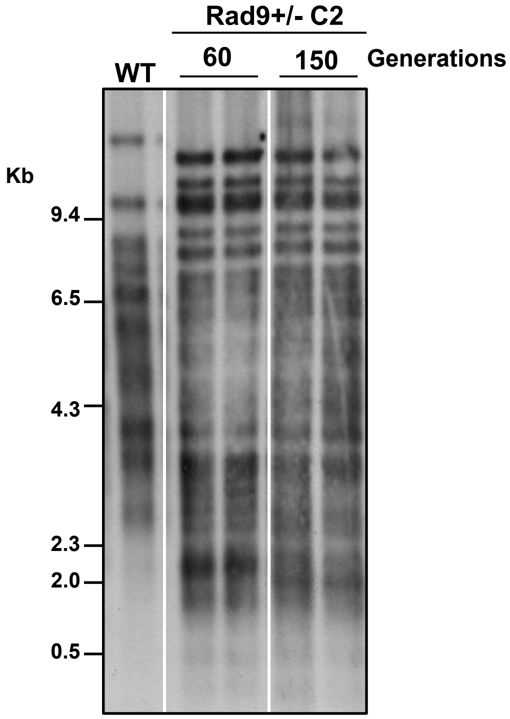

Figure S4. Analysis of telomere-containing fragments. Genomic DNA was extracted from WT cells and LmRad9+/- cells at the indicated generation in *in vitro* culture; DNA was digested with the restriction enzymes *Cvi*QI, *Hpa*II, *Alu*I and *Hha*I and probed with the telomeric sequence; no further telomere shortening was observed beyond 60 passages.

Supplementary Figure 5

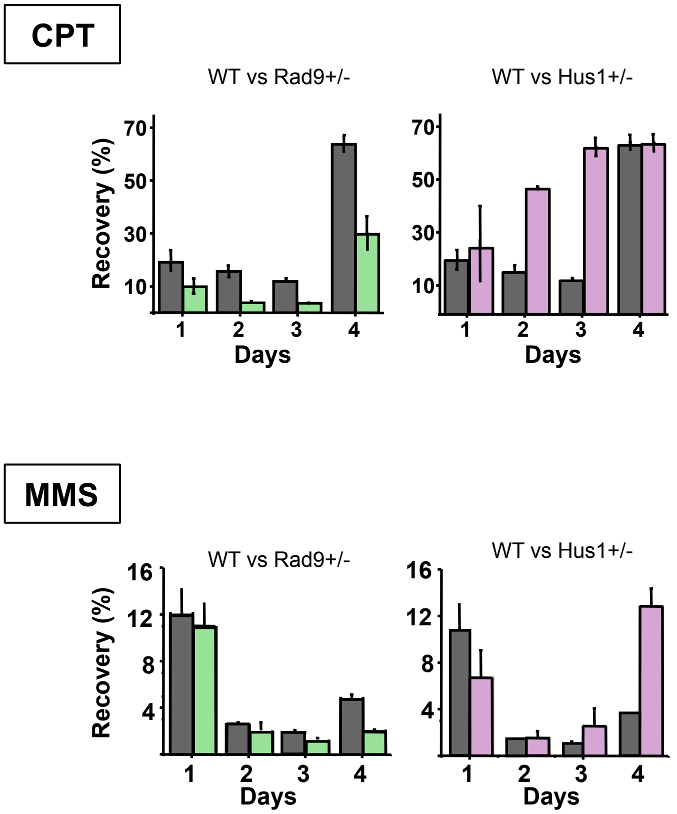

Figure S5. Recovery profile of LmRad9 and LmHus1 deficient cells upon CPT of MMS exposure. WT (gray), LmRad9+/- (green) and LmHus1+/- (purple) cells were treated with 10 μM CPT (upper panels) or 0.005% MMS (bottom panels) for ~15 hours and seeded in drug-free fresh media at 10^5 cells/mL; cell densities were assessed daily and recovery was calculated as a percentage of non-treated cells; vertical lines on top of each bar indicate standard deviation.

Supplementary Figure 6

A

	Experiment	
	**#1**	**#2**
**WT**	206	247
**Rad9+/-**	235	222
**Hus1+/-**	220	253

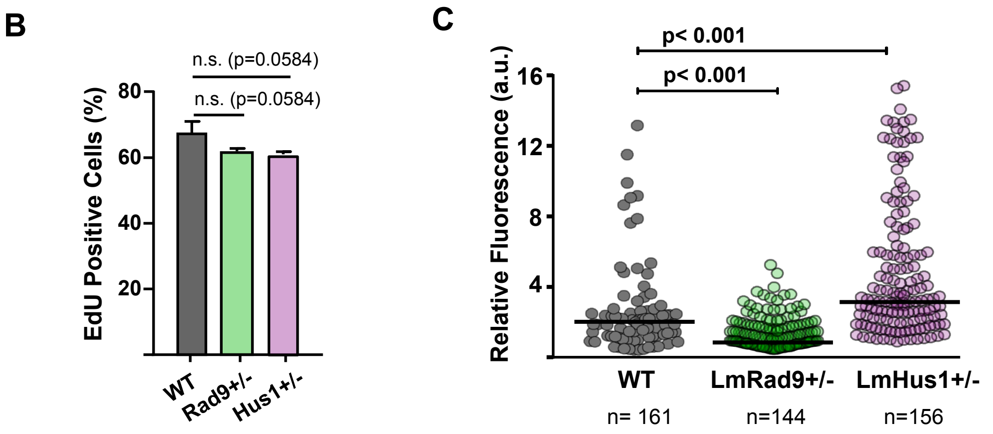

Figure S6. Analysis of EdU incorporation in WT, LmRad9+/- and LmHus1+/- cells. A) Numbers indicate total cells analyzed for each cell line in two independent experiments. **B)** Average EdU-positive cells from experiments #1 and #2; vertical lines on top of each bar indicate standard deviation; the differences in the percentage of incorporation between the tested cell lines was considered as not significant (n.s.), as determined by ANOVA analysis; *p* value is indicated.
C) Graphical representation of EdU incorporation as measured in arbitrary units (a.u.); each dot represent fluorescence intensity of an individual EdU-positive cell; horizontal bars indicate the average fluorescence intensity; n indicates the number of EdU-positive cells analysed for each cell line; *p* value as determined by Kruskal-Wallis test is indicated

Type of analysis= Anova

Supplementary Figure 7

A

L. Major	**H2A**	**107**	GVVPNISKAMAKK-KGGKKGKATPSA	**131**
T. brucei	**H2A**	**108**	GVMPSLNKALAKKQKSGKHAKATPSV	**133**

B

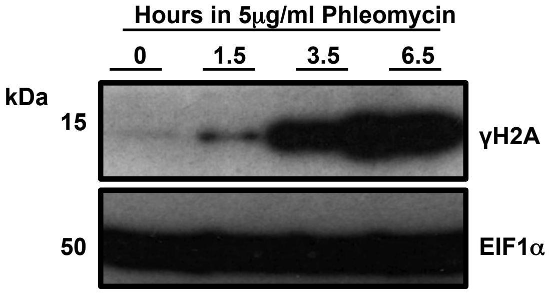

Figure S7. Testing anti-TbγH2A antibody for *Leishmania major*. A) Alignment of the *C*-termini of histone H2A sequences from *L. major* (LmjF29.1720) and *T. brucei* (Tb827.7.2820) generated by ClustalW2; aminoacid residues functionally conserved between sequences are represented with the same color; the Threonine residue, which is phosphorylated in TbH2A, is conserved in LmH2A and it is highlighted with a box. **B)** Total cell extracts from *L. major* WT cells was prepared after incubation with Phleo by the indicated period of time; extracts were probed with anti-TbγH2A antibody; the same membrane was also probed with anti-EF1α antibody to serve as a loading control.

Supplementary Figure 8

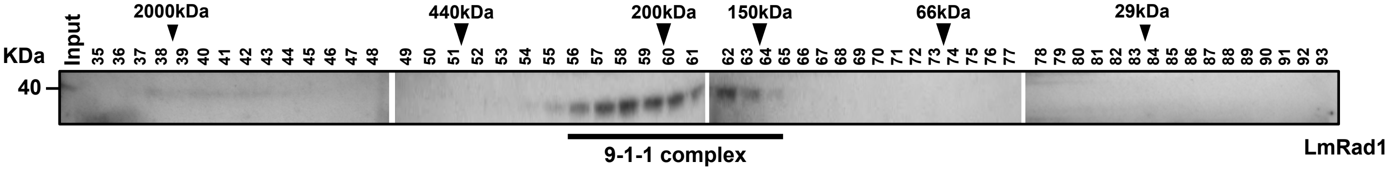

Figure S8: Western blot analysis of WT cell extract fractionated by gel filtration. Total cell extract from WT cells was subjected to fractionation in a Superdex 200 column; the indicated fractions (numbers above each lane) were probed with anti-LmRad1 antibody; arrowheads indicate peak elution fractions for calibration standards: dextran blue (2000 Kda), apoferritin (440 KDa), β-amylase (200 KDa), alcohol dehydrogenase (150 KDa), bovine serum albumin (66 KDa) and carbonic anhydrase (29 Kda).
